# Perfectionism and a Healthy Attitude toward Oneself: Could Humor Be a Resource?

**DOI:** 10.3390/ijerph17010201

**Published:** 2019-12-27

**Authors:** Annamaria Di Fabio, Martin M. Smith, Donald H. Saklofske

**Affiliations:** 1 Department of Education, Languages, Intercultures, Literatures and Psychology (Psychology Section), University of Florence, 50135 Florence, Italy; 2 School of Sport, York St. John University, York YO31 7EK, UK; m.smith3@yorksj.ac.uk; 3 Psychology Department, University of Western Ontario, London, ON N6A 3W2, Canada; dsaklofs@uwo.ca

**Keywords:** perfectionism, humor styles, well-being, psychology of harmonization, psychology of sustainability and sustainable development, strengths-based preventative perspectives

## Abstract

In the framework of the psychology of harmonization, the present study analyzes the relationships of humor styles with perfectionism, controlling for the effects of personality traits. One hundred and forty-eight Italian university students were administered the Italian versions of the HEXACO-60, the Humor Styles Questionnaire, and the short-form version of the Big Three Perfectionism Scale. Based on hierarchical regression analyses, humor styles accounted for a significant percentage of incremental variance beyond personality traits in relation to three major perfectionism factors. Humor styles may be a promising area for further research and intervention in relation to managing issues related to perfectionism in strengths-based preventative perspectives.

## 1. Introduction

Among the 17 goals for sustainability and sustainable development [1], well-being and happiness are related to the third goal (good health and well-being, to ensure healthy lives and promote well-being for all). In this framework, the psychology of harmonization [2] represents a point of reference for a new approach in the psychology of sustainability and sustainable development [3,4,5]. The aim of the tpsychology of sustainability and sustainable development is to identify and foster new strengths from a primary preventative perspective [6,7,8], promoting health and well-being in the natural environment and in different environments. This study further examines one’s personal environment from a strengths-based preventative perspective [9], identifying new resources to foster healthy attitudes toward oneself but that may be challenged by the belief that one needs to be perfect.

### 1.1. Perfectionism

Perfectionism is defined as a personal characteristic that involves striving for excellence and setting extremely high performance standards for oneself accompanied by excessively critical evaluations of one’s own actions [10,11,12]. Perfectionism is best conceptualized in the literature as a multidimensional personality disposition [13], and various measures of multidimensional perfectionism have been proposed. After the Frost Multidimensional Perfectionism Scale (FMPS) [10], the most noticeable and commonly used measure is the Hewitt–Flett Multidimensional Perfectionism Scale (HF-MPS) [11]. The publication of the new Big Three Perfectionism Scale (BTPS) [12] reflects recent advances in perfectionism research [12,14]. The BTPS was developed to supply a more in-depth analysis of three key factors in a multidimensional description of perfectionism. The BTPS [12] is a self-report questionnaire, composed of 45 items, which enables the assessment of three global perfectionism factors: rigid perfectionism, self-critical perfectionism, and narcissistic perfectionism including 10 core perfectionism facets. Rigid perfectionism is a self-oriented rigid insistence that one’s own performance must be perfect and impeccable, feeling worthwhile only if and when perfect. Self-critical perfectionism relates to having excessively negative reactions to perceived failures, worries about performance, and being severely self-critical when performance is not perfect. Narcissistic perfectionism concerns the propensity to have excessive expectations of others, cruel devaluation of others and their inadequacies, and the beliefs that some individuals deserve special treatment and consider themselves to be perfect or superior to others. The BTPS [12] has opened new and promising perspectives for research and intervention because it is the first and only scale that measures narcissistic perfectionism [15]. More recently, Di Fabio et al. [15] constructed an Italian short-form version of the BTPS, comprising 18 items while retaining the three-factor structure. More recently, Feher et al. [16] developed a 16 item short form of the BTPS replicating the three factor structure. Having a short version of this scale offers a valuable opportunity for research where participants have a large battery of questionnaires to complete within a limited time [17].

Further to the study of perfectionism is the need [12] to determine how perfectionism relates to current models of personality. Examining the research literature, the following associations emerged [13] between the well-known perfectionism dimensions of Hewitt and Flett’s [11] model and the Five Factor model of personality: Self-oriented perfectionism showed primarily a positive relationship with conscientiousness and secondarily with extraversion; other-oriented perfectionism showed a negative relationship with agreeableness but a positive relationship with neuroticism; socially prescribed perfectionism was positively correlated with neuroticism and negatively with extraversion and agreeableness. However, there has been less research with the HEXACO model of personality (19). Stoeber [18] presented the following associations regarding the perfectionism dimensions of Hewitt and Flett’s [11] model: Self-oriented perfectionism was positively associated with conscientiousness; other-oriented perfectionism was inversely associated with honesty–humility and with agreeableness; socially prescribed perfectionism was inversely associated with honesty–humility.

A recent study of the full form BTPS [12] showed the following relationships with the Five Factor model of personality: rigid perfectionism was positively associated with conscientiousness and neuroticism; self-critical perfectionism was positively correlated with neuroticism and inversely with extraversion and agreeableness; narcissistic perfectionism was positively associated with neuroticism and inversely with agreeableness. Using the BTPS-SF [16], rigid perfectionism was positively associated with neuroticism and negatively with agreeableness; self-critical perfectionism was positively associated with neuroticism and inversely with extraversion, conscientiousness and agreeableness; and higher narcissistic perfectionism was associated with lower agreeableness and with openness.

The only study [16] using the BTPS-SF and the HEXACO model of personality showed: Rigid perfectionism had a small positive relationship with conscientiousness and small inverse relationships with honesty–humility, extraversion, and agreeableness; self-critical perfectionism showed a medium inverse relationship with extraversion, a small positive relationship with neuroticism/emotionality, and small inverse relationships with agreeableness and honesty–humility; narcissistic perfectionism showed a medium inverse relationships with honesty–humility and agreeableness and small negative correlations with conscientiousness and openness to experience.

### 1.2. Humor

From a strengths-based perspective [19,20,21] directed at increasing resources to manage risks and foster healthy attitudes toward oneself [6,7,8,22], humor and humor styles have emerged in the literature as a promising new approach. Humor styles are conceptualized in different ways: As a cognitive ability (e.g., ability to create, understand, reproduce, and remember jokes [23]); an aesthetic response (e.g., humor appreciation, enjoyment of particular types of humorous material [24]); a habitual behavior pattern (e.g., tendency to laugh frequently, to tell jokes and amuse others, to laugh at others’ jokes [25]); an emotion-related temperament trait (e.g., habitual cheerfulness [26]); an attitude (e.g., bemused outlook on the world, positive attitude toward humor [27]); a coping strategy or defense mechanism (e.g., tendency to maintain a humorous perspective in the face of adversity [28]).

Martin, Puhlik-Doris, Larsen, Gray, and Weir [29] described four kinds of humor styles labelled affiliative humor, self-enhancing humor, aggressive humor, and self-defeating humor. Affiliative humor involves sharing humor with others, telling jokes and funny stories, amusing others, making others laugh, and enjoying laughing along with others. Self-enhancing humor describes a person who maintains a humorous outlook on life even when not with others, uses humor in coping with stress and to and cheer oneself up. Aggressive humor is the tendency to use humor to disparage, put down, or manipulate others; use of ridicule, offensive humor; compulsive expression of humor even when inappropriate. Self-defeating humor reflects amusing others at one’s own expense, self-disparaging humor; laughing along with others when being ridiculed or put down; and using humor to hide one’s true feelings from oneself and others. 

Relationships between humor styles and maladaptive outcomes such as burnout have emerged. In the study by Tümkaya [30] of university lecturers, positive relationships for both aggressive humor and self-defeating humor and burnout were found, whereas inverse relationships emerged for both affiliative humor and self-enhancing humor with burnout. In a study of physicians [31], higher severity of burnout symptoms was correlated with less frequent utilization of adaptive humor styles (self-enhancing and affiliative). A recent study by Ho [32] on burnout among secondary school teachers found that high affiliative and self-enhancing humor were associated with lower emotional exhaustion and depersonalization.

Using the Italian short-form version [19] of the BTPS [12], this study investigates the relationship between perfectionism and the HEXACO personality model [19] and further explores the relationship of humor styles with perfectionism dimensions, controlling for the effects of personality traits.

## 2. Aim and Hypotheses

The present study examined the relationships of the BTPS-SF perfectionism dimensions (rigid, self-critical, narcissistic) with the HEXACO personality traits. It also analyzed the relationships of the perfectionism dimensions with humor styles, controlling for the effects of personality traits (HEXACO model).

Based on the only study in the literature [16], the following three hypotheses were formulated:

**Hypotheses 1** **(H1).** *Rigid perfectionism will show a positive relationship with conscientiousness and inverse relationships with honesty–humility, extraversion, and agreeableness;*

**Hypotheses 2** **(H2).** *Self-critical perfectionism will have an inverse relationship with extraversion, agreeableness and honesty–humility and a positive relationship with neuroticism/emotionality;*

**Hypotheses 3** **(H3).** *Narcissistic perfectionism will be inversely related to honesty–humility agreeableness, conscientiousness and openness to experience.*

The following three other research questions, H4, H5, and H6, are exploratory because prior research has not considered these aspects.

**Hypotheses 4** **(H4).** *Humor styles will each add varying amounts of additional variance to that accounted for by the HEXACO personality traits in predicting rigid perfectionism.*

**Hypotheses 5** **(H5).** *Humor styles will each add varying amounts of additional variance to that accounted for by the HEXACO personality traits in predicting self-critical perfectionism.*

**Hypotheses 6** **(H6).** *Humor styles will each add varying amounts of additional variance to that accounted for by the HEXACO personality traits in predicting narcisstic perfectionism.*

## 3. Method

### 3.1. Participants

One hundred and forty-eight Italian university students attending different schools at the University of Florence participated in the study. Participants included 36% men and 64% women (mean age = 22.73 years, SD = 2.17).

### 3.2. Measures

HEXACO-60: The HEXACO-60 [19] Italian version [33] is composed of 60 items rated on a 5-point Likert scale ranging from 1 = Absolutely false to 5 = Absolutely true. Each of the six personality dimensions is assessed with 10 items. Cronbach alpha coefficients are: 0.78 for Honesty/Humility, 0.79 for Emotionality, 0.78 for Extraversion, 0.76 for Agreeableness, 0.77 for Conscientiousness, and 0.78 for Openness [33].

Humor Styles Questionnaire: The Humor Styles Questionnaire (HSQ) [29] using the Italian version (Di Fabio, 2019) is composed of 32 items answered on a 7-point Likert-type scale (from 1 = totally disagree to 7 = totally agree). The questionnaire permits the individuation of four humor styles: Affiliative humor (example of item “I enjoy making people laugh”), self-enhancing humor (example of item “It is my experience that thinking about some amusing aspect of a situation is often a very effective way of coping with problems”), aggressive humor (example of item “When telling jokes or saying funny things, I am usually not very concerned about how other people are taking it”), Self-defeating humor (example of item “I let people laugh at me or make fun at my expense more than I should”). Cronbach alpha coefficients are reported to be 0.88 for affiliative humor, 0.83 for self-enhancing humor, 0.80 for aggressive humor, and 0.79 for self-defeating humor [34].

Big Three Perfectionism Scale: The BTPS [12] Italian short-form version [15] is composed of 18 items rated on a Likert scale from 1 = Strongly agree to 5 = Strongly disagree. The BTPS-SF taps three global perfectionism factors: Rigid perfectionism, self-critical perfectionism, and narcissistic perfectionism. Rigid perfectionism includes items of two facets of the original version: Self-oriented perfectionism (example of item: “I have a strong need to be perfect” and self-worth contingencies (example of item: “I could never respect myself if I stopped trying to achieve perfection”). Self-critical perfectionism includes items of four facets of the original version: Concern over mistakes (example of item: “The idea of making mistakes frightens me”), doubts about actions (example of item: “I feel uncertain about most of my actions”), self-criticism (example of item: “I have difficulty forgiving myself when my performance is not flawless”), and socially prescribed perfectionism (example of item: “People are disappointed in me whenever I don’t do something perfectly”). Narcissistic perfectionism includes items of four facets of the original version: Other-oriented perfectionism (example of item: “I expect those close to me to be perfect”), hypercriticism (example of item: “I get frustrated when other people make mistakes”), entitlement (example of item: “I am entitled to special treatment”), and grandiosity (example of item: “I am the absolute best at what I do”). Cronbach alpha coefficients are: 0.83 for rigid perfectionism, 0.88 for self-critical perfectionism, and 0.83 for narcissistic perfectionism [15].

### 3.3. Procedure and Data Analysis

The questionnaires were administered to the university students in small groups by trained psychologists. The order of administration was counterbalanced to control the possible effects of the presentation of the instruments. The instruments were administered according to the Italian legal requirements for privacy and informed consent. Correlations and hierarchical regressions were calculated for all measures. However, multiple correlations and the potential for multicollinearity within and between personality and also perfectionism factors require that these significance values be viewed as very conservative estimates. While Bonferroni corrections may be applied, more informative is an examination of the reported coefficients in the following tables using Cohen’s effect size criteria. 

## 4. Results 

Correlations between the HEXACO-60, HSQ, and BTPS are reported in Table 1. 

Using Cohen’s [35] criteria for small, medium, and large effect sizes, rigid perfectionism showed a large positive relationship with conscientiousness and a medium inverse relationship with honesty–humility; self-critical perfectionism showed a medium inverse relationship with extraversion and a small positive relationship with honesty–humility; narcissistic perfectionism showed a medium inverse relationship with honesty–humility and low inverse relationships with emotionality and agreeableness.

For rigid perfectionism as a dependent variable, the overall model explained 42% of the variance. Personality traits (HEXACO) accounted for 15% of the variance and humor styles added another 27% with the largest contribution from aggressive humor (Table 2).

For self-critical perfectionism as a dependent variable, the overall model explained 46% of the variance. Personality traits (HEXACO) explained 22% of the variance with humor styles adding an additional 24%, the self-defeating style followed by affiliative humor contributing most to this relationship (Table 3).

For narcissistic perfectionism, the overall model explained 47% of the variance. Personality traits (HEXACO) explained 15% of the variance. Humor styles added 32% of the variance with the largest contribution from aggressive humor (Table 4).

## 5. Discussion

The present study examined the relationships between perfectionism (rigid, self-critical, narcissistic) personality traits (HEXACO model) and humor styles (affiliative, self-enhancing, aggressive, and self-defeating) including an analysis of the relationships of the combined effects of personality and humor styles to perfectionism. 

The first hypothesis was partially confirmed. In the present study, rigid perfectionism showed a positive relationship with conscientiousness and an inverse relationship with honesty–humility, but these relationships were stronger than those in the study by Fehr et al. [16]. The correlation with conscientiousness was large (0.62) and the correlation with honesty–humility was medium, whereas these correlations were small in the study by Fehr et al. [16]. Furthermore, in the present study, unlike in the study by Fehr et al. [16], inverse relationships did not emerge between rigid perfectionism and both extraversion and agreeableness. 

The second hypothesis was also partially confirmed. Self-critical perfectionism showed a medium inverse relationship with extraversion and a small positive relationship with honesty–humility, as in the study by Fehr et al. [16]. In contrast to the study by Fehr et al. [16], inverse relationships did not emerge between self-critical perfectionism and both emotionality and agreeableness. 

The third hypothesis was also partially confirmed. Narcissistic perfectionism showed a medium inverse relationship with honesty–humility and low inverse relationships with emotionality and agreeableness. Compared to Fehr et al. [16], the present study suggested that narcissistic perfectionism had a low inverse association with emotionality and not with conscientiousness and openness to experience. Furthermore, the correlation with agreeableness was low in the present study, whereas in the study by Fehr et al. [16], it was medium.

The final three analyses showed that humor styles explained a significant percentage of incremental variance beyond personality traits in relation to perfectionism dimensions. In particular, in relation to rigid perfectionism, aggressive humor gave the greater contribution (0.41), followed by affiliative humor (inverse association) and self-defeating humor (positive association). Perfectionist individuals who have a rigid insistence that their own performance must be perfect and impeccable, and who feel worthwhile only when perfect, seem to use primarily a humor style characterized by the tendency to disparage, put down, or manipulate others [29]. In relation to self-critical perfectionism, self-defeating humor offered the greater contribution followed by affiliative humor (inverse association). In particular, perfectionist individuals with a propensity to have excessively negative reactions to perceived failures, and the propensity to become involved in severe self-criticism when their performance is not perfect, appear to use a humor style characterized by a tendency to amuse others at one’s own expense and, to a lesser extent, with a humor style characterized by the tendency to share humor with others, tell jokes and funny stories, and amuse others [29]. As expected, narcissistic perfectionism was most highly related to aggressive humor (0.46) followed by affiliative humor (inverse relationship) and self-defeating humor (positive association). Perfectionist individuals who show a propensity to have excessive expectations of others, and tend towards more cruel devaluation of others and their inadequacies, appear to use more aggressive humor styles [29]. It is noteworthy that, in this study, the humor styles offered the greater contribution, in particular aggressive humor, to narcissistic perfectionism and rigid perfectionism. In relation to self-critical perfectionism, the greater positive contribution was offered by self-defeating humor but also observed in the other two perfectionism factors, albeit to a lesser extent. As expected, affiliative humor showed inverse relationships with all the three dimensions of perfectionism in contrast to self-enhancing humor styles that did not show any association. This means that the humor style characterized by the tendency to share humor with others, make others laugh, and enjoy laughing along with others does not appear to be involved in relation to the three dimensions of perfectionism. In summary, this study showed promising empirical relationships between humor styles and perfectionism dimensions beyond those simply accounted for by major personality factors. If these relationships are confirmed in future studies, humor styles could be considered for their impact toward further understanding intrapersonal attitudes as well as attitudes and behavior towards others.

It is necessary to underline some limitations to this study. The data analysis method in this study assumes no measurement error, and thus results could be over-interpreted. Furthermore, the participants were a limited to a small group of Italian university students, and thus are not representative of all Italian university students. Future research should, therefore, extend the study of the relationship between these variables to other groups of university students and the wider public in Italy and other countries and cultures. It would also be interesting to replicate this study and extend the research to include other measures with other individual strength characteristics such as gratitude [36] and self-compassion [37]. 

## 6. Conclusions

The results of the present study, if confirmed, andgiven that humor styles may be more amenable to training in contrast to personality traits, would add support to developing pro-social humor styles as a positive resource. Thus, humor styles could be seen as personal strengths and a promising area for further research and intervention [9] in relation to perfectionism. In the framework of the Psychology of Harmonization [2] and the psychology of sustainability and sustainable development [3,4,5], humor styles could represent promising new factors for enhancing the well-being of individuals and for fostering healthy attitudes toward oneself.

## Figures and Tables

**Table 1:** Standard deviations, and correlations between HEXACO-60, Humor Styles Questionnaire (HSQ), and Big Three Perfectionism Scale (BTPS).

Variable	1	2	3	4	5	6	7	8	9	10	11	12	13
1. HEXACO-60 Honesty–humility	-												
2. HEXACO-60 Emotionality	0.20 *	-											
3. HEXACO-60 Extraversion	−0.02	−0.30 **	-										
4. HEXACO-60 Agreeableness	0.20 *	0.30 **	−0.18 *	-									
5. HEXACO-60 Conscientiousness	0.14	0.16	0.18	−0.07	-								
6. HEXACO-60 Openness to experience	−0.14	−0.01	0.12	−0.08	0.17 *	-							
7. Affiliative humor style	−0.22 **	−0.12	0.45 **	−0.04	0.11	−0.31 **	-						
8. Self-enhancing humor style	−0.28 **	−0.29 **	0.29 **	0.07	−0.14	0.19 *	−0.17 *	-					
9. Aggressive humor style	−0.20 *	−0.37 **	0.14	−0.29 **	−0.26 **	−0.17*	−0.04	−0.01	-				
10. Self-defeating humor style	−0.36 **	−0.01	−0.27 **	0.08	−0.14	0.21 *	−0.06	0.15	0.28 **	-			
11. Rigid perfectionism	−0.30 **	−0.14	0.06	0.01	0.62 **	−0.10	−0.21 *	−0.12	0.50 **	0.29 **	-		
12. Self-Critical perfectionism	−0.27 **	0.18 *	−0.30 **	0.01	−0.15	0.15	−0.29 **	−0.09	0.12	0.58 **	0.56 **	-	
13. Narcissistic perfectionism	−0.32 **	−0.23 **	0.09	−0.17 *	−0.16	−0.01	−0.17 *	−0.01	0.57 **	0.40 **	0.82 **	0.60 **	-

Note: *N* = 148. ** *p* < 0.01. * *p* < 0.05.

**Table 2:** The contributions of personality traits HEXACO-60 (first step) and Humor Styles (second step) to rigid perfectionism.

Personality Traits and Humor Style	Rigid Perfectionism
	Β
Step 1	
HEXACO-60 honesty–humility	−0.28 **
HEXACO-60 emotionality	0.01
HEXACO-60 extraversion	0.13
HEXACO-60 agreeableness	−0.03
HEXACO-60 conscientiousness	0.22 **
HEXACO-60 openness to experience	−0.04
Step 2	
Affiliative humor style	−0.29 **
Self-enhancing humor style	−0.07
Aggressive humor style	0.41 ***
Self-defeating humor style	0.14 *
R^2^ step 1	0.15 ***
∆R^2^ step 2	0.27 ***
R^2^ total	0.42 ***

Note: *N* = 148. * *p* < 0.05. ** *p* < 0.01. *** *p* < 0.001; Standardized coefficients are shown in the above table.

**Table 3:** The contributions of personality traits HEXACO-60 (first step) and Humor Styles (second step) to self-critical perfectionism.

Personality Traits and Humor Style	Self-Critical Perfectionism
	Β
Step 1	
HEXACO-60 honesty–humility	−0.25 **
HEXACO-60 emotionality	0.16
HEXACO-60 extraversion	−0.30 **
HEXACO-60 agreeableness	−0.02
HEXACO-60 conscientiousness	−0.04
HEXACO-60 openness to experience	−0.09
Step 2	
Affiliative humor style	−0.30 ***
Self-enhancing humor style	−0.04
Aggressive humor style	0.01
Self-defeating humor style	0.46 ***
R^2^ step 1	0.22 ***
∆R^2^ step 2	0.24 ***
R^2^ total	0.46 ***

Note: *N* = 148. ** *p* < 0.01. *** *p* < 0.001; Standardized coefficients are shown in the above table.

**Table 4:** The contributions of personality traits HEXACO-60 (first step) and Humor Styles (second step) to narcissistic perfectionism.

Personality Traits and Humor Style	Narcissistic Perfectionism
	Β
Step 1	
HEXACO-60 honesty–humility	−0.22 **
HEXACO-60 emotionality	−0.04
HEXACO-60 extraversion	0.19
HEXACO-60 agreeableness	−0.03
HEXACO-60 conscientiousness	0.01
HEXACO-60 openness to experience	0.06
Step 2	
Affiliative humor style	−0.29 ***
Self-enhancing humor style	0.03
Aggressive humor style	0.43 ***
Self-defeating humor style	0.23 **
R^2^ step 1	0.15 ***
∆R^2^ step 2	0.32 ***
R^2^ total	0.47 ***

Note: *N* = 148. ** *p* < 0.01. *** *p* < 0.001; Standardized coefficients are shown in the above table.
